# Decoding Cancer Evolution: Integrating Genetic and Non-Genetic Insights

**DOI:** 10.3390/genes14101856

**Published:** 2023-09-24

**Authors:** Arghavan Ashouri, Chufan Zhang, Federico Gaiti

**Affiliations:** 1 Princess Margaret Cancer Centre, University Health Network, Toronto, ON M5G 2M9, Canada; 2 Department of Medical Biophysics, University of Toronto, Toronto, ON M5G 1L7, Canada

**Keywords:** cancer evolution, genetic alterations, epigenetics, tumour heterogeneity, clonal expansions, multicellularity, single cell, phenotype, plasticity, tumour microenvironment

## Abstract

The development of cancer begins with cells transitioning from their multicellular nature to a state akin to unicellular organisms. This shift leads to a breakdown in the crucial regulators inherent to multicellularity, resulting in the emergence of diverse cancer cell subpopulations that have enhanced adaptability. The presence of different cell subpopulations within a tumour, known as intratumoural heterogeneity (ITH), poses challenges for cancer treatment. In this review, we delve into the dynamics of the shift from multicellularity to unicellularity during cancer onset and progression. We highlight the role of genetic and non-genetic factors, as well as tumour microenvironment, in promoting ITH and cancer evolution. Additionally, we shed light on the latest advancements in omics technologies that allow for in-depth analysis of tumours at the single-cell level and their spatial organization within the tissue. Obtaining such detailed information is crucial for deepening our understanding of the diverse evolutionary paths of cancer, allowing for the development of effective therapies targeting the key drivers of cancer evolution.

## 1. Introduction

Cancer is a complex disease characterized by the accumulation of cellular abnormalities over time [1,2,3,4,5]. Throughout the years, several pivotal models have shaped our comprehension of this phenomenon. More than a century ago, Theodor Boveri laid the groundwork for our understanding of the origin of malignant tumours. He postulated that cancers stem from genetic alterations in normal cells and introduced the concept that most tumours and their metastases originate from a single cell, influencing the contemporary notion of clonal expansion [6]. The Armitage and Doll model, established in the 1950s, emphasized the role of multiple genetic changes over time in transforming healthy cells into malignant ones [7]. This model proposed that cancer development is a multistep process characterized by a series of sequential cellular abnormalities, requiring six to seven successive mutations in affected cells. The cumulative impact of mutations on cancer growth was demonstrated in landmark research on retinoblastoma in 1971, motivating the Knudson’s “two-hit” concept [8]. In the late 1970s, Peter Nowell pioneered the integration of evolutionary concepts to comprehend cancer’s origins and evolution, whereby Darwinian key evolutionary principles (variation, heredity, and selection [9]) were applied to elucidate the mechanisms responsible for cancer formation and development [10]. Nowell’s model suggested that most tumours arise from a single neoplastic cell and evolve through a process of selection for somatic alterations, leading to the proliferation and survival of the most fitted clones [10]. Dynamic heterogeneity, proposed by Harris and colleagues in the early 1980s, highlighted the emergence of metastatic clones from genetically or epigenetically diverse cell populations [11]. This concept illuminated the intricate dynamics of cancer evolution and underscored the significance of tumour heterogeneity. In the 1990s, Fearon and Vogelstein proposed the “multi-hit” model of cancer. It contended that cancer arises due to the accumulation of multiple genetic mutations in normal cells, and these mutations are tied to the histological traits of these tumours [12]. This model further promoted the notion of cancer’s multistage progression and provided key insights into tumour suppressors and oncogenes.

Altogether, these influential models and concepts have significantly advanced the understanding of cancer progression mechanisms, paving the way for a Darwinian framework in modeling tumour evolution and therapy resistance [1,13,14,15,16,17]. However, it is important to note that individual tumours may not conform to a single evolutionary model, and multiple mechanisms may operate simultaneously or at different stages of progression. Recent evidence indicates significant macroevolutionary leaps in cancer, involving rapid accumulation of genetic abnormalities due to events such as chromosomal instability, chromoplexy, and chromothripsis [18,19,20,21,22,23]. Additionally, oncogenes amplification within extrachromosomal DNA during replication has been identified as a common event in cancer, with unique inheritance patterns [24,25,26,27,28,29]. Recently, there has been an increasing recognition of the fact that tumour evolution is not solely driven by genetic alterations, but also influenced by non-genetic factors, such as cell plasticity and tumour microenvironment (TME) [4,30,31,32,33,34,35,36].

In this review, we explore these concepts and advocate for an inclusive approach considering both Darwinian and non-Darwinian patterns in cancer evolution. We explore the mechanisms by which unicellularity and multicellularity become decoupled during cancer’s onset and development. Lastly, we emphasize the significance of advancements in omics technologies in the study of cancer evolution.

## 2. Models of Cancer Evolution: From Linear Succession to Punctuated Equilibrium

**Figure 1:**
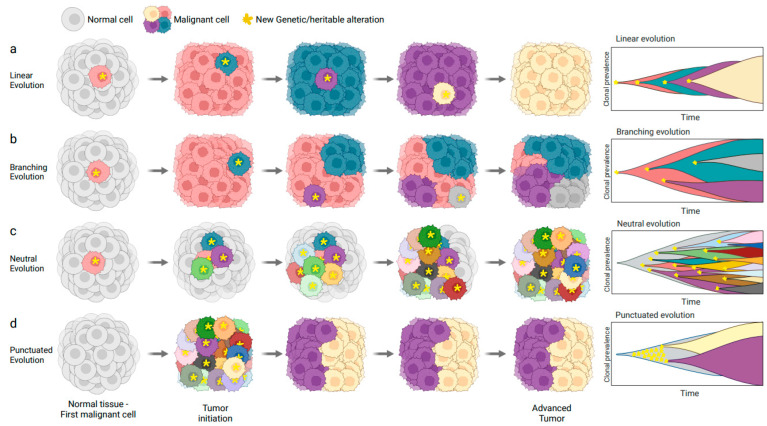
Models of cancer evolution shaped by genetic alterations. (a–d) Depiction of four distinct models illustrating the progression of cancer influenced by genetic factors. The cellular representation (**left panel**) demonstrates the evolution at the individual cell level, highlighting cells impacted by genetic changes. The evolutionary trajectory view (**right panel**) pictures the pathway or progression of cancer evolution over time due to genetic alterations. In both panels, cells that have accumulated a new genetic alteration are indicated with a yellow star.

Currently, our comprehension of tumour evolution encompasses a diverse array of models, with at least four well-recognized paradigms: linear, branching, neutral, and punctuated, as depicted in Figure 1 [37]. Traditionally, cancer progression was perceived as an orderly procession of clonal cell divisions, where genetic modifications accumulate in precursor cells in a step-by-step manner, providing a substantial selective advantage to these cells. This advantageous shift eventually leads to the dominance of newer clones (Figure 1a). Consequently, tumours were believed to contain clonally identical cells because of ongoing cycles of selective sweeps. Initial studies on tumour evolution adhered closely to this linear model; however, the advent of advanced genomic technologies, notably next-generation sequencing (NGS), catalyzed a transformative shift in the field [38,39]. More recently, the use of single-cell sequencing has ushered in a new era of understanding, allowing for the detection and analysis of intratumour heterogeneity (ITH), which is explained by the coexistence of molecularly and phenotypically distinct subclones within a tumour [4]. This scenario supports an alternative model, proposing that tumour growth occurs in a non-linear, branched pattern. In this model, various subclones, stemming from a common ancestor, diverge and proliferate simultaneously, each with varying levels of fitness [1,40] (Figure 1b).

While these models account for accumulating genetic changes influenced by selective pressure, they prove inadequate in incorporating the full spectrum of cancer’s evolutionary dynamics. In fact, according to the neutral tumour evolution model [41], cancer-driving alterations arise not due to selective advantages but owing to the random fixation of selectively neutral mutations through genetic drift (Figure 1c). Consequently, the ITH observed in tumours primarily emerges from the stochastic fixation of nearly neutral changes within the population, lacking a functional role in promoting tumour growth. Lastly, several lines of evidence suggest that, in some cases, genetic aberrations in cancer cells may occur in short bursts of time [18,19,20,21,22,23,42]. The punctuated equilibrium model embodies this phenomenon, suggesting that tumour cells undergo extended periods of relatively stable mutational rates interspersed with short spans of intense evolution. During these bursts, tumour cells can accumulate multiple driver events, contributing to the intricate patterns of cancer evolution [43] (Figure 1d).

Overall, our current understanding of tumour evolution has evolved from a linear clonal progression model to encompass a diverse spectrum of paradigms, including branching, neutral, and punctuated patterns. These models, shaped by selective pressure, random mutations, and bursts of intense evolution, collectively contribute to the complex dynamics of cancer growth and ITH, highlighting the need for the integration of these multifaceted evolutionary patterns in our quest to decode cancer evolution.

## 3. Cancer Initiation and Progression as a Reverse Microevolutionary Process

While each of the evolutionary models presented above makes a different assumption regarding when the mutations occur and how clones navigate through the selective pressure, what is clear is that in most cases, tumours stem from alterations occurring in a single cell or a small cluster of cells within a larger multicellular ecosystem, such as the human body. Comparable to multicellularity and the underlying cooperation between cells which have independently evolved several times [44], the evolution of cancer occurs repeatedly. Thus, each individual tumour represents a unique occurrence of an evolutionary process.

Within the framework of multicellular organisms, a multitude of regulatory pathways actively suppress the fitness of individual cells, thereby safeguarding the overall fitness of the organism [45]. Adhesion proteins were one of the earliest components that facilitated the organisms to transition from uni- to multicellular. These proteins played a pivotal role in fostering coherence between neighboring cells [46]. This process led to enhanced interaction and synchronization among diverse cell types, as well as the specialization of various cell lineages within an organism, resulting in improved adaptability and overall fitness gain [47]. This phenomenon is often described as a shift from prioritizing individual cell fitness to favoring the fitness of the entire organism. Interestingly, the very regulatory mechanisms that evolved to ensure effective cell interactions have also introduced vulnerabilities. When these finely tuned cellular regulations are disrupted, they can create conditions conducive to the development of malignant tumours [48]. Many recognizable traits of cancer can indeed be traced back to the disruption of molecular networks that were established during the evolutionary emergence of multicellularity [49,50]. A multitude of characteristics exhibited by cancer cells remarkably mirror those of their unicellular counterparts. These encompass sustained signals for cell proliferation, evasion of programmed cell death, and the acquisition of unlimited replicative potential, among other traits [51].

In essence, the onset of cancer can be viewed as a form of reverse microevolution, a process whereby cancer cells regress from the multicellular agreement and evolve towards a state reminiscent of unicellular organisms [51,52,53]. The interplay between the driving forces of multicellular evolution and the complex pathways of cancer development underscores the profound interconnectedness of these biological phenomena.

## 4. Diverse Dimensions in Cancer Evolution: Beyond Genetic Drivers

**Figure 2:**
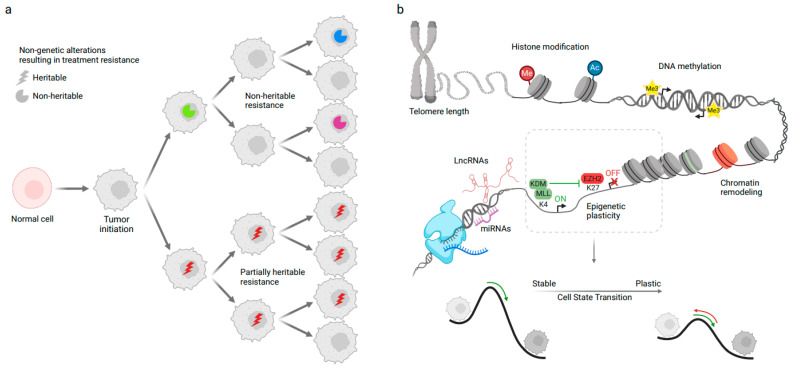
Cancer evolution and the non-genetic factors involved in treatment resistance. (**a**) Non-genetic factors can be either heritable (partially) or non-heritable. In case of non-heritable factors, some cells can gain the beneficial state for resistance, but they cannot pass it on to the next generation of cells (**top**). The heritable states might follow a heritable path, although the degree of heritability is lower than genetic factors. (**b**) **Top**: Examples of intrinsic factors causing non-genetic changes in the cell. Only one example of the regulators of cell plasticity is shown (the H3K27 methyltransferase EZH2, the H3K4 methyltransferase MLL, and the lysine demethylase KDM). **Bottom**: A zoomed-in representation of the cell state changes caused by non-genetic factors. These changes lower the barriers between malignant cells, increasing cell state plasticity.

The application of models inspired by Darwinian evolutionary principles has provided a suitable framework for studying cancer evolution [16,54]. In fact, human somatic cells take part in an evolutionary process characterized by continuous changes, selection, and the growth of cell clones. In recent years, pervasive somatic mutations have been identified across a plethora of healthy tissues, suggesting that cancer often arises from premalignant clonal outgrowths [55,56,57,58,59,60,61,62,63,64], as in the context of clonal hematopoiesis. In this process, cells accumulate various alterations, most of which are passenger events without functional impact [65]. However, occasionally, clones of cells carrying specific alterations can take over healthy tissues, leading to the development of preneoplastic lesions and malignant tumours [66]. In this scenario, a single cell might acquire a new heritable genetic or non-genetic alteration that increases its chances of producing a viable offspring, often referred to as a selective advantage (Figure 1 and Figure 2). These cells then outcompete their counterparts that are lacking the advantageous alteration, leading to clonal expansion [1]. As this process unfolds, the growing population continues to diversify through additional alterations and undergoes positive selection [36,54,67,68]. This diversity supports the notion that cancer is not a singular entity; rather, numerous distinct subpopulations exist within each tumour. This increasing ITH presents a grand challenge for effective cancer treatment, increasing the odds of both pre-existence of tolerant and resistant subpopulations [69]. As the population of cancerous cells further enlarges, so does its clonal diversity. This diversity furnishes the tumour with a vast array of alterations, some of which may confer adaptability to therapy [70,71]. Consequently, even with the advancements in therapeutic approaches, many malignant tumours adapt rapidly and return in a more aggressive and resilient form [72]. To address this challenge, it is key to determine the functional significance of ITH and trace the evolution of diverse cancer cell clones. Such insights will shed light on the specific subsets of cells that drive disease progression. Moreover, genetic and non-genetic drivers identified during cancer evolutionary trajectories have the potential to serve as critical biomarkers for early disease monitoring and assist clinical decision making [73,74]. This could result in the development of alternative treatment strategies to prevent tumours from advancing to life-threatening stages.

Current NGS data challenge the conventional perspective that cancer progression and resistance solely stem from genetic alterations. In fact, only a minority of genetic variants have been identified as responsible drivers for tumour progression, metastasis, immune evasion, and therapy resistance [75,76]. An approach by which tumours can survive treatment is for some cells to randomly (stochastically) enter a treatment-resistant state, in a process not mediated by heritable, genetic mechanisms [33,77,78,79] (Figure 2a). However, recent studies also point to an alternate route: heritable mechanisms that are non-genetic (Figure 2a), which facilitate cancer evolution allowing persistence or lineage plasticity [75,80]. As evidence, lung cancer cells have demonstrated non-genetic heritable resistance to treatment when subjected to therapeutic inhibition of the EGFR protein [76]. Similarly, melanoma cells can adopt specific drug-tolerant transcriptional profiles that show a degree of heritability [81,82]. Lastly, even in cancer cell populations with largely identical genetic backgrounds, a significant degree of plasticity, a phenomenon in which a cell can switch states in a dynamic and reversible manner, is observed [31,83]. Cancer cell plasticity can be triggered by, among other factors, heterogeneity at either transcriptomic or epigenomic level or both, resulting in changes in survival, stemness potential, and proliferative capability. For instance, in a recent study on lung cancer, a high-plasticity cell state was identified. These cells revealed high capacity of differentiation, proliferation, and resistance to chemotherapy, suggesting that this high-plasticity state might have a role in driving disease aggressiveness and progression [73].

Recent breakthroughs, especially in single-cell sequencing technologies (discussed in the next section), have shown extensive intratumoural variability in cell states, epigenetic profiles, spatial dynamics, alternative splicing patterns, and tumour microenvironment interactions [34,55,56,84]. These non-genetic modifications are crucial contributors of cancer cell phenotypes. In fact, human cells possess mechanisms that allow inheritable phenotypic changes in the absence of DNA changes. Epigenetic identities can be reliably perpetuated from the initial altered cell, permitting inference of the cell of origin, like the faithful propagation of the cancer cell’s genetic information. Consequently, just as stochastic errors in the genome can lead to genetic variability and diversification within tumours, similar errors in the epigenetic makeup might result in a diverse epigenetic landscape within the tumour (Figure 2b). This blurs the lines between epigenetic identities and key non-genetic factors that drive cancer’s progression [84,85,86,87,88,89]. Telomere lengthening, addition of methyl groups (methylation) to DNA, changes to the DNA-binding histone proteins, binding of various microRNAs (miRNAs) and long non-coding RNAs (lncRNAs), and modifications to the chromatin structure are some key examples of epigenetic changes with ample evidence supporting their heritability, as well as their contribution to the pathobiology of malignancies (Figure 2b, top) [90,91,92,93,94,95,96]. For instance, previous studies, based on bulk bisulfite sequencing, have identified thousands of loci with a “noisy” stochastic pattern of DNA methylation changes. These changes indicate significant heritable variations in the epigenome of both normal and cancerous tissues [84,85,86,87,88,89]. In addition, changes that disrupt chromatin can lead to overly permissive chromatin states, lowering barriers, ultimately resulting in increased cell plasticity (Figure 2b, bottom) [97]. As cancers progress, this epigenetic variation amplifies, leading to a loss of distinct epigenetic identity. This enhances the evolutionary potential of the cancer, often resulting in unfavorable outcomes. A high degree of DNA methylation can also inactivate genes that aid in tumour suppression. A well-known example of such a process is the methylation and silencing of the promoter region of *MGMT* gene, a O-6-methylguanine-DNA methyltransferase. Methylation of this gene drives a hypermutator phenotype that generates many genetic subclones in the tumour [98], thus suggesting that epigenetic alterations might precede and directly lead to genetic changes [97].

The coexistence of genetic and non-genetic factors driving cancer evolution has also been observed in patient samples. Additionally, the evolutionary pressures and selection processes that shape the growth and progression of tumours can differ widely, even within a single type of cancer [72,99,100]. A specific study on triple-negative breast cancer exemplifies this: whole exome and RNA sequencing of autopsy tissues demonstrated that distinct metastatic lesions had evolved independently [101]. Some lesions had different mutations in drug target regions, consistent with the higher mutation rate observed in this subtype of breast cancer. Yet, other lesions displayed unique gene expression patterns without any detectable key mutations. This indicates that even within a single primary tumour, multiple evolutionary pathways might be at play. Therefore, for effective cancer treatment, it is crucial to consider this complexity and distinguish between functional changes and inconsequential ones, regardless of whether they originate from genetic or non-genetic factors. However, in some cases, there is consistency in the evolutionary trajectories between genetic and non-genetic factors. For instance, a multiregion study of lung adenocarcinomas found that the tumour evolution patterns inferred from both somatic copy number alterations and DNA methylation were vastly comparable [102]. Similar congruencies in genomic and epigenomic evolution have been observed in papillary renal cell carcinoma [103].

Cancer cells might also inherit transcriptional plasticity and epigenetic memory from their initial cell state, which may contribute to their ability to adapt within the tumour environment, including developing resistance to treatments and the potential to metastasize (Figure 2a) [75,104,105,106]. Indeed, current research indicates that the cancer-enabling phenotypes, such as persistence and lineage plasticity, might be encoded and propagated epigenetically [34,97,106,107,108]. Additionally, critical transcription factors (TFs) may facilitate the propagation of treatment-resistant cellular traits [109]. For instance, abnormal activation of embryonic pluripotency TFs including *SOX2*, *OCT4*, and *NANOG* could drive cancer cells into a stem-like state and promote further acquisition of aggressive phenotypes [110,111,112]. Other studies have shown that in prostate and breast cancer, plasticity and lineage reprogramming can be driven by activation of JAK/STAT pathway, which promotes resistance to endocrine therapy [113,114].

Resistance to cancer therapy is usually multifaceted; hence, targeted therapies might not always be effective in such circumstances. A better understanding of key functional genetic and non-genetic factors underlying tumour resistance will influence treatment strategies. For instance, over the last decade, immunotherapy has revolutionized the treatment of many tumours [115,116]. Several in-depth studies have shown that a high mutation burden, often caused by errors in DNA mismatch repair, is a strong indicator of how effective immune checkpoint inhibitors could be [117,118]. However, relying solely on the mutational burden alone does not guarantee success, as many patients do not have a long-lasting response to these treatments [119]. Moreover, mismatch repair deficiency does not always lead to immune activation in cancer [120]. Growing evidence suggests that epigenetic alterations might be the basis for individual variations in patient-specific drug response. Epigenetic alterations frequently exhibit high plasticity. This means they can be potentially reversible and thus represent promising therapeutic targets. Currently, the primary treatments targeting these epigenetic alterations focus on DNA methylation, modifications of DNA-associated proteins (such as histone deacetylation), and noncoding RNAs like microRNAs [121]. The incidence of multiple genetic and non-genetic aberrations in tumours suggests that combining standard treatments with those targeting epigenetic changes could lead to more effective treatments, for instance by restoring the sensitivity in resistant tumour cells by reactivating previously silenced genes [122].

Altogether, these findings highlight the need for more comprehensive models of cancer evolution. The integration of multiple layers of information from individual cancer cells is critical for fully grasping the mechanisms driving cancer progression and to better understand how genetic and non-genetic factors contribute to cancer progression and resistance. Addressing these challenges can greatly benefit from multiomics technologies, enabling the capture of simultaneous layers of information at the single-cell level—the fundamental unit of cancer evolution [4].

## 5. Integrating Single-Cell and Spatial Multiomics for Comprehensive Insights into Cancer Evolution

The advent of next-generation sequencing (NGS) techniques has significantly propelled our comprehension of the molecular compositions of cancer cells [123,124,125,126]. However, a substantial portion of these data has been garnered from molecules extracted from bulk tumour samples, a heterogeneous mix of malignant and non-malignant cells, each playing pivotal roles in tumour progression and resistance [127].

**Figure 3:**
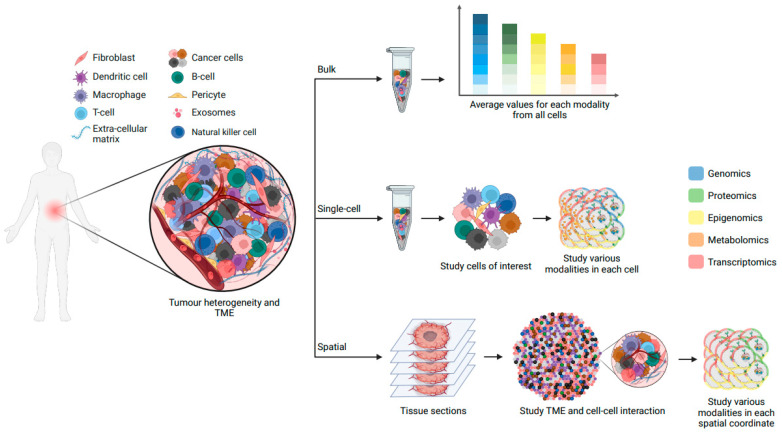
An overview of the power of single-cell and spatial multiomics techniques for studying intratumour heterogeneity and TME. Using bulk sequencing methods, different modalities (shown in five colours of blue, green, yellow, orange, and red) can be studied in each cell type, but only an average value per sample is measured. Single-cell technologies allow the study of different modalities within each individual cell simultaneously. Using spatial omics, the interactions between cancer cells as well as TME can be studied in a spatial context.

Cells, as the fundamental constituents of multicellular organisms, exhibit remarkable diversity in morphology and function throughout development and disease. Typically, cells are categorized into discrete “types” or “states” based on traits such as gene expression, morphology, and functionality. Single-cell RNA sequencing (scRNA-seq) has revolutionized this classification paradigm by measuring gene expression in thousands—even millions—of individual cells [127]. This technology has facilitated finer-grained identification of cell types, subtypes, and states within dynamic and complex cancer systems. Recent studies have highlighted transcriptional cell state diversity across tumour types that is often independent of genetic heterogeneity [128,129,130]. For instance, in brain tumours, several distinct malignant cell states have been identified, with some associated with higher stemness potential (neural progenitor-like and oligodendrocyte progenitor-like cells) and others associated with a more differentiated state (astrocyte-like and mesenchymal-like cells) [131,132,133,134,135,136]. However, the transcriptome is only one component of a cell’s phenotype, and it is an incomplete representation of cellular identity. In fact, molecular and cellular identity emerge from the interplay of numerous cellular modalities, all of which can fluctuate due to internal and external factors [137]. Alongside the widespread adoption of scRNA-seq, novel single-cell multiomics sequencing methods have emerged, enabling the simultaneous assessment of multiple factors influencing ITH and cancer evolution (Figure 3). These factors encompass clonal heterogeneity, intratumoural differentiation programs, tumour microenvironment (TME), and spatial organization, as well as metastasis and resistance mechanisms.

As a cancer cell population evolves, cells accumulate genetic and non-genetic alterations that trigger the emergence of new clones with distinct, selective advantages (see Section 4). It is therefore essential to connect these alterations to cellular phenotypes or functions, to ultimately unveil functional drivers of advantageous clonal outgrowths. Single-cell multiomics methodologies allow for the integration of genotypic data—ranging from single nucleotide variations to whole chromosome alterations—with epigenomic, transcriptomic, and proteomic data [4,138,139]. For instance, we recently developed a method for the concurrent profiling of gene expression, surface proteins, somatic mutations, and RNA splicing in individual cells [56]. By utilizing this method, we investigated the effects of mutations in genes encoding RNA splicing factors in patients with myelodysplastic syndrome and clonal hematopoiesis, unveiling splicing abnormalities that lead to lineage-specific clonal expansions. On another end, epigenetic remodeling also underpins the plasticity that enables cancer cells to switch between different states at various disease stages, including tumour initiation, metastasis, and development of therapy resistance [97]. By employing multiomics methods that capture epigenome, transcriptome, and genomic alterations within the same cell, we and others have characterized patterns of DNA methylation, chromatin accessibility, and gene expression in single glioma cells. These methods reveal epigenomic underpinnings of cellular heterogeneity and plasticity, including mechanistic insight into cellular transitions between stem-like and differentiated-like states, suggesting that epigenetic dysregulation contributes to the maintenance of stemness features [34,108,140]. These single-cell multiomics approaches highlight instances where non-genetic factors, rather than genetic mutations, drive cancer progression and resistance to therapies ([32,33]; also reviewed in [4]).

The propensity of certain cancer cells to leave their Initial location and infiltrate distant organs, also known as metastasis, is the leading cause of cancer-related mortality [50]. From its transformation to its settlement on a new tissue, the metastatic cancer cell undergoes significant modifications, such as adopting a greater motility program (epithelial-to-mesenchymal transition), evading immune cell monitoring, and adjusting to the new secondary location [141,142]. ScRNA-seq coupled with lineage tracing technologies applied to non-small-cell lung cancer tumours has revealed a striking diversity in cells’ ability to metastasize. Interestingly, this heterogeneity was shown to have arisen from pre-existing, heritable transcriptional cell states [143]. By applying a similar approach on pancreatic cancer metastasized tumour, a rare epithelial-to-mesenchymal transitioning state was discovered that showed the highest metastatic potential [144]. Dynamic epigenetic processes appear to govern pivotal stages of metastasis, as no single mutation or set of mutations stands out as reliable predictors of metastatic behavior across tumour types [145,146]. For instance, in lung adenocarcinoma, single-cell epigenomic profiling revealed a chromatin state continuum in the transition toward metastatic state and defined a premetastatic accessibility program characterized by activation of RUNX transcription factors [147]. Regarding therapy resistance, undetected by bulk techniques due to their rarity, certain malignant subclones harbor critical genetic and non-genetic alterations conferring resistance to treatments. These subclones often dominate after initial therapy, triggering recurrence. Several single-cell studies have proven that in multiple cancer types, cells that survived chemotherapy were often derived from minor clones in sensitive populations, and that therapy resistance is the result of both pre-existing non-genetic features and subsequent cell state transitions [32,75,105,148,149].

Although single-cell multiomics methods have allowed us to explore the complex interplay between genetic and non-genetic determinants of cancer evolution, it is important to recognize that the full understanding of a cancer cell’s identity hinges not only on its molecular makeup but also on its life history and spatial context within the tumour tissue. As opposed to being randomly distributed within the tumour, malignant and non-malignant cells occupy specific niches within the tumour space, leading to defined cell–cell interactions. These microenvironmental interactions have a direct impact on cancer development by profoundly altering the transcriptome and epigenome of both cancerous and neighboring non-cancerous cells [150], as well as by promoting clonal selection. For example, in clonal hematopoiesis, during chronic inflammation, the production of proinflammatory cytokines has been shown to promote the growth of mutated hematopoietic stem cells (HSCs) [151,152,153,154]. Further, single-cell spatial mapping of cellular composition and spatial organization of the primary and metastatic brain tumours’ TME has revealed that several cellular neighborhoods characterized by CD4+ T cells or M1-like macrophages are correlated with patient survival [155]. Moreover, investigations into locoregional tumours have revealed that DNA methylation patterns are intricately tied to the spatial configuration of colorectal cancer cells [156]. The diversity in DNA methylation profiles in such cases has been linked to better outcomes such as relapse-free survival or a higher overall survival rate.

Recent advances in spatial methods based on DNA, RNA, multiplexed fluorescence, and isotope labelling have provided insights into cellular composition of tissues while preserving spatial information [157]. These techniques have aided in unraveling tumour architecture and microenvironmental interactions. For example, in glioblastoma samples, a recent study employed spatially resolved multiomics to identify distinct niches characterized by immunological and metabolic stress factors. These spatial niches were shaped by the tumour microenvironment and showcased transcriptional adaptations to inflammatory or metabolic stimuli, mirroring neural developmental stages [158]. Further, the emergence of spatial multiomics approaches, which integrate the detection of mRNA and proteins [159], or mRNA and epigenomic patterns [138,160,161], has introduced a new dimension to our understanding of cancer progression (Figure 3). These methodologies offer unprecedented glimpses into clonal development, the intricate interplay between cancer cells and the tumour microenvironment, and the regional diversity within the tumour. This essential information might illuminate non-genetic mechanisms behind cancer progression and treatment resistance, such as faulty regulatory systems that let cancer cells evade the immune system.

The non-genetic mechanisms discovered to drive cancer evolution could pave the way for new therapeutic strategies. For pre-existing features that confer cancer cells’ resilience against chemotherapy agents, treatment combination against both proliferation- and resistance-related targets could be administrated simultaneously and be more effective in eliminating the tumour. For instance, simultaneous treatment with specific epigenetic agents (e.g., KDM6i and 5FU) prevented H3K27me3 demethylation and entry into drug-tolerance states, delaying tumour relapse [162]. Additionally, research on epithelial-to-mesenchymal transition (EMT), a key non-genetic regulator in cancer progression, showed the effectiveness of an anti-netrin-1 antibody (NP137) in inhibiting tumour growth and enhancing chemotherapy sensitivity in endometrial carcinomas. This has led to promising early-stage clinical trials [163,164]. Furthermore, in the TME, studies have indicated the role of tumour-associated macrophages in promoting tumour growth, with the STAT3 pathway being a key player [165]. Immunotherapeutic agents targeting this pathway are currently being tested in clinical trials (e.g., NCT03382340), showing promising response rates in certain cancer patient groups.

In sum, emerging single-cell and spatial multiomics techniques provide an unprecedented opportunity to not only decipher the genetic and non-genetic determinants of cancer but also to offer transformative prospects for novel therapeutic strategies. Further multiomics advances promise insights into the coordinated regulation of individual cells, crucial for understanding holistic cellular phenotypes in development, health, and cancer therapy.

## 6. Conclusions and Perspectives

In conclusion, studying cancer progression and resistance has undergone a remarkable evolution, driven by the interplay of innovative models and technologies that have expanded our understanding of cancer complexity. From Boveri’s foundational genetic alterations concept to the multi-hit model of Fearon and Vogelstein, and Peter Nowell’s integration of evolutionary principles, these models have significantly contributed to our insights into cancer origin and development. However, the complexity of cancer evolution extends beyond these models, encompassing a spectrum of paradigms including linear, branching, neutral, and punctuated patterns. These patterns underscore the multifaceted nature of cancer evolution, influenced by both genetic and non-genetic factors.

While genetic mutations remain a cornerstone of cancer evolution, non-genetic mechanisms have gained prominence as key contributors to ITH, resistance to therapy, and metastatic potential. Recent advancements in omics technologies, particularly single-cell and spatial techniques, have unveiled the intricate interplay between genetic and non-genetic determinants, revealing the importance of epigenetic modifications, transcriptional plasticity, and microenvironmental interactions in shaping cancer cell identity and behavior. The incorporation of spatial context further enriches our understanding by shedding light on the interactions between cancer cells and their surroundings. In this context, the concept of cancer evolution mirrors the fundamental transition from unicellularity to multicellularity, as cancer cells seem to regress from the collaborative state of multicellular organisms to a more individualistic, unicellular-like behavior. This reverse microevolution underscores the intricate interplay between the forces that drive multicellular evolution and the pathways that govern cancer development.

It is evident that embracing a comprehensive approach that integrates both genetic and non-genetic determinants in the study of cancer evolution is essential. This entails integrating Darwinian and non-Darwinian patterns of evolution and recognizing the impact of diverse mechanisms, from genetic mutations to non-genetic modifications and microenvironmental interactions. As our knowledge of cancer evolution expands, it is vital to harness technological advancements in multiomics methodologies, in conjunction with spatial analysis, to decipher the coordinated regulation of individual cancer cells. This will ultimately enhance our capability to predict tumour behavior, identify subclones resistant to treatment, and pave the way for innovative therapeutic strategies that directly anticipate and address cancer evolution.
